# Exploring expectations and readiness for healthy lifestyle promotion in Swedish primary health care: a qualitative analysis of managers, facilitators, and professionals

**DOI:** 10.1080/02813432.2023.2301556

**Published:** 2024-02-07

**Authors:** Emma Nilsing Strid, Lars Wallin, Ylva Nilsagård

**Affiliations:** a University Health Care Research Center, Örebro University, Örebro, Sweden; b School of Health and Welfare, Dalarna University, Falun, Sweden

**Keywords:** Qualitative research, implementation science, leadership, primary health care, health personnel, healthy lifestyle, practice guidelines

## Abstract

**Objective:**

Prior to a multifaceted implementation strategy for a healthy lifestyle-promoting practice the expectations of primary health care managers, appointed internal facilitators and health care professionals on supporting change was explored.

**Design:**

This study had an explorative qualitative design using data gathered from individual interviews and focus groups. Qualitative content analysis with a deductive category development was applied using the Consolidated Framework for Implementation Research.

**Setting and participants:**

The study was conducted in a primary care setting in central Sweden as a part of the Act in Time research project. Prior to a multifaceted implementation strategy, we held 16 individual interviews with managers and appointed facilitators and five focus groups with 26 health care professionals.

**Results:**

Managers, facilitators, and professionals held similar expectations, where their expressed need for support corresponded to three constructs: Readiness for implementation, Implementation climate, and Engaging. Our findings indicate the need for strong leadership engagement to focus on how the healthy lifestyle-promoting practice can be anchored among the professionals. Managers at all levels should communicate the vision and goals, enable facilitators and professionals to improve their competencies, build inter-professional teams, and jointly plan the new practice.

**Conclusion:**

To change to a healthy lifestyle promoting practice professionals request support from their managers, who in turn need support from the middle and top managers. The requested support includes helping to prioritise health promotion and enabling the primary care centres to build competence and take ownership of the implementation.

**Trial registration:**

ClinicalTrials.gov NCT04799860

## KEY POINTS

- This study revealed that primary health care managers and health care professionals had mutual expectations of supporting a change into a healthy lifestyle-promoting practice before implementation.
- Strong leadership engagement was perceived as essential across all manager levels, including assistance in prioritising health promotion.
- The support should enable primary health care centres to build competence and have ownership of implementing a healthy lifestyle-promoting practice.

## Introduction

Non-communicable diseases continue to account for 80% of the overall burden of disease in Europe [1]. Modifiable behavioural risk factors such as tobacco use, physical inactivity, unhealthy diet and harmful use of alcohol all increases the risk of non-communicable diseases [2]. Several initiatives have been taken to address these challenges and increase the quality of care across OECD countries [3], including Sweden [4]. Many of these initiatives share common features in the leading paradigm ‘integrated care,’ proposed as the future direction for developing healthcare systems worldwide [5–8]. Cornerstones in integrated care focus on persons and relationships, coordination, co-production, and proactive and health-promoting strategies [4,8]. Thus, primary care is crucial in this shift towards integrated care, such as encouraging healthier lifestyles [9]. However, applying the knowledge of the association between unhealthy lifestyles and non-communicable diseases (e.g. diabetes, cardiovascular diseases, and respiratory diseases) [1,10] is a huge challenge for health care professionals (HCPs) [11–15].

In 2018, clinical practice guidelines for health promotion and disease prevention were established in Sweden, targeting unhealthy lifestyle habits, including tobacco use, harmful use of alcohol, low physical activity, and poor nutrition [16]. However, there has been limited use of the guidelines [17], suggesting that HCPs may need more support to change current practices. The *Act in Time* research project was initiated to evaluate the process and outcomes of a multifaceted implementation strategy for a healthy lifestyle-promoting practice using individually targeted lifestyle interventions in Swedish primary health care (PHC) [18]. One central component in the 12-month implementation strategy are external facilitators (EFs) and internal facilitators (IFs), as described in the i-PARIHS framework [19,20]. Another is steps in the change leadership model [21,22] with a focus on intrinsic motivation, i.e. ‘the doing of an activity for its inherent satisfaction rather than for some separate consequence’ [23]. When designing methods to change the behaviour of HCPs, the use of theory and involving end users have been proposed to adapt and tailor the implementation strategy to the needs of the HCPs [24]. In this study the Consolidated Framework for Implementation Research (CFIR) [25] was chosen as the theoretical framework to explore determinants for implementing a health promotion practice before starting implementation support. This approach has been suggested but is less used in pre-implementation phases [26–29]. In the *Act in Time* research project the perceptions of PHC managers and HCPs on barriers and facilitating factors for implementing a healthy lifestyle-promoting practice have been described [30]. Moreover, the support PHC managers and HCPs think they need to overcome the identified barriers and use the facilitating factors to change into a healthy lifestyle-promoting practice needs further research. The collected data provides a basis for the implementation strategy and enables the selection and adaption of implementation activities [31–33] and may thereby contribute to a successful implementation strategy.

### Aim

This study explored the expectations of PHC managers, appointed IFs, and HCPs on support of a change to a healthy lifestyle-promoting practice.

## Material and methods

### Design

An explorative qualitative design was chosen to collect data from individual interviews [34] and focus groups [35] at a pre-implementation stage. Qualitative content analysis was applied with a deductive category development [36].

### Setting and recruitment

As part of the *Act in Time* research project, the current study was conducted in a PHC setting in central Sweden [18]. The intervention centres were five PHC centres, representing variation in size, socio-economic status, and geographic and location (in both geographic location and urban vs. rural). At each centre, PHC managers appointed two HCPs as IFs, expected to support the implementation of change in close collaboration with the managers and EFs. At the pre-implementation stage, we invited first-level PHC managers, appointed IFs, and HCPs at these centres to participate in individual interviews (managers and IFs) and focus groups (HCPs). The invitation, including information about the study and participation, was sent by e-mail to the managers and the appointed IFs. All of those who had been invited agreed to participate. At each PHC centre, the manager or one of the appointed IFs forwarded the invitation to participate in a focus group to their co-workers, i.e. HCPs at their centre. We sought to include a diversity of professionals (e.g. counsellors, general practitioners, nurses, and physiotherapists) in each group. We conducted five focus groups with 4-7 HCPs in each group and individual interviews with 6 managers and 10 appointed IFs. Participant characteristics are provided in Table 1.

**Table 1.** Description of participant characteristics.

Participants	Age, mean (min–max) years	Sex, Men/Women	Profession	Working years within primary care, mean (min–max) years
Individual interviews				
Managers (*n* = 6)	47.8 (40–55)	1/5	1 Care administrator, 2 district nurses, 1 Midwife, 2 Physiotherapists,	15.7 (5–28)
Appointed internal facilitators (*n* = 10)	43.9 (31–58)	0/10	3 District nurses, 1 Midwife, 2 Nurses, 1 Occupational therapist, 3 Physiotherapists	9.35 (1.5–33)
Focus groups				
A (*n* = 7)	48.4 (30–59)	2/5	1 Counsellor, 2 District nurses, 1 Midwife, 2 General practitioners, 1 Physiotherapist	10.4 (6 months −27)
B (*n* = 4)	41.5 (34–50)	0/4	1 Assistant nurse, 1 Counsellor, 1 Midwife, 1 Physiotherapist,	5.3 (4–7)
C (*n* = 4)	58.2 (49–63)	0/4	1 Counsellor, 2 District nurses, 1 Paediatric nurse,	12.8 (2–22)
D (*n* = 6)	50.3 (25–65)	1/5	1 Assistant nurse, 1 Care Administrator, 1 Counsellor, 1 District nurse, 1 General practitioner, 1 Physiotherapist	13.7 (3–36)
E (*n* = 5)	42.2 (26–49)	2/3	1 Counsellor, 1 District nurse, 2 General practitioners, 1 Physiotherapist	5.2 (3–9)

### Data collection

For the individual interviews and focus groups, we developed and pilot-tested three slightly different semi-structured interview guides based on CFIR constructs [25]. The CFIR includes five major domains: Innovation Characteristics, Outer Setting, Inner Setting, Characteristics of Individuals, and the Implementation Process. Each domain contains several of constructs [25]. The constructs in CFIR describe contextual factors that may impact implementation processes, particularly barriers and facilitators that may influence efforts to integrate and sustain change in clinical practice [37]. A selection of CFIR constructs is often used in data collection and data analysis [28]. The interview questions in this study focused on constructs related to engagement and the need for support within the Inner Setting and Implementation Process domains (see Appendix A for the interview guides). All questions were open-ended, and the participants were encouraged to speak freely and from their experiences. Questions were complemented with probes (e.g. ‘can you tell me more,’ ‘could you explain that’ and ‘what do you mean’), loops, and transitions to gain more in-depth knowledge. Because of the restrictions during the COVID-19 pandemic, most individual interviews were conducted by phone or digitally (Visiba Care), but all focus groups were held face-to-face at the PHC centres. Data were collected from April 2021 to February 2022 by one author (ENS). Another author (YN) participated as an observer in the focus groups, taking field notes and observing the interaction and discussion flow, and to ask complementary or clarifying questions. Each focus group took 53–69 (mean 61) minutes and individual interviews 35–67 (mean 45) minutes. The focus groups and individual interviews were digitally recorded and transcribed verbatim by two authors (ENS and YN) or a professional transcriber. The transcribed texts were imported into Nvivo 12 (QSR International, Melbourne, Australia) to manage and code the data.

### Data analysis

Data were analysed using a deductive qualitative content analysis [36] guided by the CFIR theoretical framework [25]. The data analysis began when the interviews and focus groups were held at each PHC intervention centre. The two authors responsible for the analysis (ENS and YN) read all transcripts to acquire a general understanding of the data. For the deductive coding, we developed a structured categorisation matrix based on the domains and constructs in the CFIR [25] (see Appendix B). ENS and YN coded together two transcripts using the CFIR constructs. Discussions on the accuracy of the construct coding were held with a third author (LW). Thereafter, three additional transcripts were jointly coded to reach a shared understanding of the data, CFIR constructs, and the coding strategy. ENS then coded the remaining transcripts. Consensus discussions among all authors were held twice to ensure that the meaning units were coded into the most appropriate constructs of the CFIR. Depending on the content of the meaning unit and the context that the participants were talking about, the data could be coded in another construct than was referred to in the interview guide (Appendix A).

The codes concerning support were deductively categorised into constructs within the CFIR domains Inner Setting and Implementation Process. Thereafter, different categories were created within the CFIR constructs following the principles of inductive content analysis [36]. This categorisation aimed to describe the participants’ need for support more in depth, and what would enable them to change to a healthy lifestyle-promoting practice. Codes from the three groups (HCPs, managers, and IFs) were analysed separately. Thus, the results focus on what is similar and different in the narratives of these three groups. Finally, all authors discussed the categorisation and agreed upon the final version of the content and the category and subcategory labels. An example of the data analysis process is presented in Table 2. Quotations capturing the essence of the data are provided to illustrate the categories [38]. The quotations were translated into English and then backtranslated into Swedish to ensure consistency of meanings.

**Table 2.** Example of the data analysis process.

Quote	Category	CFIR sub-construct	CFIR construct	CFIR domain
We may have to schedule in another way, so they feel that they have the time and possibility to discuss this [lifestyle habits]	Allowing flexible working schedules to overcome time barriers	Available resources	Readiness for implementation	Inner setting
We need to improve our competence within this field. I realised (…) that there were quite many clichés that I thought were true. And yeah, I don’t think I’m the only one with little knowledge; instead, we all need more knowledge to dare have these conversations	Clarify why the change is needed and let us improve our competence	Access to information and knowledge		

### Ethical considerations

The Swedish Ethical Review Authority approved the *Act in Time* project (DNRs 2020-06956, 2021-00912, and 2021-05825-02). The *Act in Time* project is registered in ClinicalTrials.gov NCT04799860; https://clinicaltrials.gov/ct2/show/NCT04799860. Participants in interviews and focus groups received written and oral information about confidentiality, participant rights, and the project’s aim. There was no participant-researcher relationship. All participants provided written informed consent. The study complied with the ethical principles of the Helsinki Declaration [39]. The authors confirm that all methods were performed in accordance with relevant guidelines and regulations. The COREQ (COnsolidated criteria for REporting Qualitative research) checklist was used to conduct and report this study [40].

## Results

The narratives of PHC managers, appointed IFs and HCPs on their expectations of support for the change to a healthy lifestyle-promoting practice were related to three constructs within the two CFIR domains Inner setting and Implementation Process: Readiness for implementation, Implementation climate, and Engaging [25]. Readiness for implementation concerns the participants’ expectations of support from their managers, support to overcome barriers with limited resources, and the knowledge and competencies needed to execute a healthy lifestyle-promoting practice. Support to prioritise and the role of organisational incentives are included in the Implementation climate. Engaging includes the engagement of appointed IFs and key stakeholders and the need for support from EFs. The categories relating to the expectations of the participants are described in the coding matrix (Appendix B). We have compared and contrasted the narratives of the three groups of participants and present the comparisons in Tables 3 and 4.

**Table 3.** Readiness for implementation – the perspectives of PHC managers, appointed internal facilitators and health care professionals.

PHC managers	Internal facilitators	Health care professionals
Leadership engagement supporting the implementation of health promotion		
Top management should provide PHC managers support and communicate health promotion as a PHC commission.	All management levels should show that health promotion is important and part of routine practice.	Managers must show that health promotion practice is important and prioritised.
Show support by communicating the vision: health promotion needs to be carried out.	Managers should expect health promotion from us HCPs.	Managers must clarify this is what we do and what they expect from us.
Health-promoting practice requires participation from all HCPs and is therefore mandatory.	Managers should emphasise that health-promoting practice is mandatory.	Managers should clarify individual responsibility to participate in the health-promoting practice. The practice should be mandatory.
Provide time to plan and prepare.	Need time to plan and prepare.	Need time to plan and prepare.
Provide education, tools, and follow-up goals.	Expect to work as a team with managers to push, remind, follow up, provide feedback, and acknowledge HCPs’ efforts and ideas.	Warrant opportunities to learn more for professional development and improved competence.
Available resources – resources supporting the change into a health promotion practice		
Considering minor changes in working schedules to support and solve tight working schedules and lack of personnel.	Need to feel free to change work schedules to have time to discuss lifestyle behaviour change with patients.	Staff shortage and high workload require more flexible working schedules and more time to discuss lifestyle with patients.
Access to information and knowledge supporting the change into a health promotion practice		
All HCPs need to understand the intentions of the health-promoting practice.	All HCPs need to know why they engage in a health-promoting practice.	HCPs need to understand and relate to why they engage in a health-promoting practice.
All HCPs need rapid and updated knowledge of lifestyle habits and motivational interviewing.	HCPs’ competence varies and increased knowledge of lifestyle habits and skills in motivational interviewing is warranted.	Uncertain to raise questions on lifestyle habits because of insufficient knowledge. Managers should offer education for all HCPs at different levels.
All HCPs need to know how to refer patients to HCPs with higher competence.	Building inter-professional teams with HCPs having expertise would increase the quality of lifestyle interventions.	Building inter-professional teams with HCPs having expertise would improve the quality of lifestyle interventions.
Routines, checklists, manuals, and training would support the HCPs.	Routines, flowcharts, and checklists would support HCPs.	Routines, flowcharts, and checklists for reminders and help to provide equal care.

PHC: primary health care. HCP: health care professionals.

**Table 4.** Implementation climate and engaging for the health-promoting practice – the perspectives of PHC managers, appointed IFs and health care professionals.

PHC managers	Internal facilitators	Health care professionals
Relative priority of the health-promoting practice		
Need for clarified goals regarding the PHC commission on health promotion.	Managers should communicate that health promotion is a highly prioritised commission and a work task for all HCPs.	Need to clarify goals on prevention and health promotion with other goals, such as accessibility to PHC.
Need for top management support in prioritising health promotion and integration into ordinary routines.	Health promotion needs a higher priority. If understaffed, health promotion most easy to omit.	Need support to prioritise health promotion among patients and work tasks.
Organisational incentives and rewards supporting the change into a health-promoting practice		
Economic incentives can enable initial prioritisation, but not in the long run.	Economic incentives may enable prioritisation.	Economic incentives can have negative consequences, such as opportunism.
Health promotion should have high priority in regulatory documents, including goals, follow-ups, and rewards.	Follow-ups and rewards are required to improve health promotion practice.	Follow-ups and rewards are needed to improve health promotion practice.
Engaging: support internal facilitators (IFs) and primary health care (PHC) managers to implement a health-promoting practice		
**PHC managers**		**Internal facilitators**
The IFs should become experts in health promotion and champions for the practice of health promotion.		Need for increased knowledge on lifestyle habits and motivational interviewing, and how to encourage and support co-workers.
Expect support from external facilitators and share ideas and experiences with other managers.		Working in pairs, support from the manager and external facilitators, and the time set for the assignment would facilitate the work of IFs.

PHC: primary health care; IF: internal facilitator; HCP: health care professional.

### Leadership engagement

The most pivotal point was to anchor health promotion among the HCPs at the PHC centres. Regardless of the role, manager, IF or HCP, leadership engagement and commitment at all managerial levels were vital to achieve the successful implementation of the healthy lifestyle-promoting practice. Changing practice would be easier to accomplish when managers at all levels show that they acknowledge the importance of health promotion and expect HCPs to work with it as part of the ordinary practice and in line with the PHC commission. Coherent manager support would strengthen the HCPs to put more emphasis on health promotion. The PHC managers described a strong commitment to health promotion and awaited collaboration with their employees. Also, they realised their leadership and support were required, especially at the beginning of the change in health practice. There was a mutual understanding of the importance of managers directing their employees and clarifying that the shift into a more health promotion practice needs to be done. Every HCP must understand why the change is required and what is expected from them in the healthy lifestyle-promoting practice.

We should all have the perception that this work is important and that it is encouraged. Otherwise, I don’t think it will ever work because if you have a feeling that the boss doesn’t think it’s so important, then you won’t do it, not as well, anyway. (IF 5)

All three groups emphasised that the healthy lifestyle-promoting practice requires participation from all HCPs, which is therefore mandatory. To achieve participation, routines must be revised to be valid for all HCPs. The participants discussed the risk of health promotion is an optional practice (as some may withdraw) and stressed that managers need to clarify individual responsibility. Ultimately, this joint participation was described to guarantee patients equal care by offering individually tailored lifestyle interventions. There was a mutual need for time to plan and prepare health promotion practices. Discussions on the health-promoting practice before the implementation work starts would enable the HCPs to be informed, participate in the preparations and education, and discuss the procedure with one another. By working as a team, the IFs and managers would encourage the HCPs, push, remind, provide support and follow-up, and acknowledge the HCPs’ efforts. The HCPs strived for professional development and improved competence, recognised by the managers’ thoughts on tools, assigning time for improvement work and educational activities.

Is it good enough or should we go deeper into it? What shall we do? And I think that if any of us, me or one of my colleagues, would like to work with lifestyle habits and health, one would be given the possibility to further education and be somebody with excellent competence within this field. (Focus group D)

### Available resources

The main obstacle to implementing the health-promoting practice was the concern that the approach would increase the workload in PHC. If true, there might be a risk that the implementation fails, and the health-promoting practice would be rejected. There were common descriptions of time constraints within an understaffed organisation where the HCPs felt overloaded with accumulated work tasks. This concern of insufficient resources conflicted with the intentions to change health practice. At some PHC centres, HCPs could previously have flexible working schedules for lifestyle interventions but feel more restrained now. To facilitate the implementation all participants suggested minor changes in working schedules to have sufficient time to discuss lifestyle habits with their patients.

Do we need to change work schedules in a way that allows us to have time to discuss these [lifestyle habits] with patients? (IF 10)

### Access to information and knowledge

All three groups highlighted that emphasis must be put on the gain of healthy lifestyle-promoting practice, which would improve the HCPs’ intrinsic motivation to change and engage in better health care. Otherwise, implementing the practice will be difficult; as a manager said, ‘squeezing it in would only result in resistance.’ The HCPs’ competencies in health promotion were thought to vary, although some (such as district nurses working with diabetes) were qualified. However, there was a mutual agreement of a lack of updated evidence-based knowledge on lifestyle habits and skills in motivational interviewing. HCPs were uncertain about how to ask patients about lifestyle habits, provide simple or advanced advice, and how to refer patients to specialists, especially regarding nutrition. Education and information were prerequisites for implementing a health-promoting practice in PHC. The HCPs suggested that basic education should be offered to all HCPs, and thereby the manager would signal the importance of health promotion, motivating and enabling the HCPs to change into a more health-promoting practice. Increased knowledge would, among other things, raise the confidence levels of HCPs in discussing lifestyle habits. Advanced education was also suggested for some HCPs, enabling them to become more specialised and competent in lifestyle interventions. The HCPs proposed that each PHC centre would have HCPs with good competence and high skills in lifestyle interventions and scheduled time to advise and follow up according to clinical guideline recommendations. Collaboration over professional boundaries should be encouraged, as HCPs perceived teamwork as a foundation for health promotion where they could understand and use each other’s competencies to accelerate change. Inter-disciplinary teams would increase the quality of advice on behaviour change of different lifestyle habits and further develop and integrate a health-promoting lifestyle. Furthermore, because patients have different prerequisites, preferences, and needs, the HCPs underlined the importance of person-centred care to build an alliance and mutual trust, be humble when discussing lifestyle habits, and provide lifestyle interventions.

Managers, IFs, and HCPs requested clear routines for the health-promoting practice. The routines should be acknowledged by all HCPs and immediately introduced to new personnel. Moreover, practical and simple tools, such as checklists, manuals, flowcharts, and cheat sheets, would also facilitate the HCPs applying health promotion strategies.

But then we must understand why, and maybe take part of current scientific papers because otherwise, it feels meaningless. Yeah, and that we all know, if alcohol problems turn up, what do we do? How? What supporting lines do you suggest? Do we have information accessible to hand out? So you feel you can manage it well. (Focus group A)

### Relative priority

Managers, IFs, and HCPs acknowledged health promotion as part of the PHC commission. However, they faced challenges in contrasting goals concerning accessibility to PHC for all citizens and quality of preventive care and health promotion. In addition, the IFs and HCPs warranted clarified commissions and managers’ guidance on giving precedence to health promotion. The HCPs discussed ethical difficulties to prioritise among patients. They were uncertain about what work tasks to reduce, pass over, or de-implement to have time for the health promotion practice. To put more effort into health promotion, they needed assistance in prioritising. But also, the managers who wanted to enhance health promotion with available resources and without major reorganisations required support from their managers.

What are our options? We, who work within PHC, should work on health promotion. It’s in our commission. And we cannot always place the blame on insufficient resources or lack of time. We should instead fulfil our commission. (Manager, PHC centre 4)

### Organisational incentives and rewards

Diverging perceptions were evident as to the gain of economic incentives. Some managers thought financial incentives could enable the precedence of health promotion in PHC.

We should put more emphasis on things that we may think are important but release. Then you have a goal to reach a certain level to receive compensation. Economic incentives, I believe, can also be good. (Manager, PHC 2)

Nevertheless, I don’t believe in that model of putting money into it. No, I believe more in informing the staff so they understand why they should do it (…) and that it feels important to do it, so you will do it. (IF 3)

The HCPs discussed the negative aspects of economic incentives. They thought it triggers opportunism (e.g. prescribing physical activity only because of monetary reimbursement) and may not change their daily practice or benefit the patients in the long run. Because of their efforts to promote healthy lifestyle habits, follow-ups were considered more important. The managers spotlighted that the health promotion practice would benefit if given high priority at all manager levels, with goals and follow-ups described in regulatory documents. High priority from all manager levers and regulatory documents were especially needed to hinder the health promotion practice from diminishing, as the intentions of the managers and HCPs may not guarantee success.

### Engaging

At each PHC centre, the managers had appointed two HCPs as IFs, which they perceived had positive attitudes towards health promotion practices. They were of different professions and ages, had mixed experiences, and were encouraging and trusted co-workers. The two IFs were thought to complement each other. Finally, they described themselves as confident in managing the assignment together with education and support. The time set for the assignment (4 h/week) was appreciated support, enabling the IFs to prioritise the work. Various creative suggestions on how they could facilitate the implementation of the health-promoting practice were delineated. These suggestions ranged from information, dialogues with co-workers, and communication on why and how to change routines to providing practical tools. The managers discussed gaining support from EFs and sharing ideas and experiences with other managers, which was essential if they met reluctance in powerful professional groups. In addition, help with structures for improvement work and follow-ups was suggested. The support was deemed valuable during the 12-month implementation period, particularly at the start.

Previously, it has been the managers deciding what we should do. And then there’s an everyday clinical life where it doesn’t work. We will get support to implement this well, and something will finally happen. It won’t be someone up there discussing it; instead, we can make it happen. (Manager, PHC centre 4)

## Discussion

The main findings of this qualitative study conducted before implementing the healthy lifestyle-promoting practice show that PHC managers, appointed IFs, and HCPs shared similar experiences and expectations of support. These findings imply a mutual view of what would enable them to change to a healthy lifestyle-promotion practice that benefits their patients. Strong leadership support and engagement were considered prerequisites from all levels (HCPs to PHC managers). The support should include help to prioritise health promotion from top management and enable the PHC centres to build competence and take control over the implementation effort.

All three groups mutually emphasised the support and engagement of managers at all levels. As suggested in implementation frameworks, leadership at different levels constitutes a key implementation determinant and positively impacts outcomes [25,41,42]. However, evidence on *how* leadership influences the implementation process is inconsistent [43,44]. Managers have been shown to support their employees during implementation by performing several roles (diffusing, synthesising, mediating, and selling), influencing the implementation climate [44,45]. The PHC managers in this study described similar functions in planning the implementation of an integrated health-promoting practice by reinforcing positive expectations and supporting their employees. The PHC managers also reported examples of behaviours previously identified in the Ottawa Model of Implementation Leadership: relational, change, task behaviours and the knowledge necessary for leaders to conduct the behaviours and facilitate the implementation process [46]. In this study PHC managers and HCPs shared the view of contrasting requirements in the primary care commission and historically low priority in prevention and health promotion concerning other commissions such as first-contact accessibility. The opposing requirements and low priority were considered barriers to a healthy lifestyle-promoting practice. To embrace the health promoting-praxis along with other clinical services, the HCPs required support from their PHC managers, who needed support from their managers and top management. There was a mutual request for managers at all levels to communicate health promotion as a highly prioritised work task and commission for PHC. Higher priority would enable the PHC managers to direct and engage their employees in changing practices. These findings are consistent with those reported by Birken et al. on how top manager support may increase middle managers’ commitment to the implementation of innovations [47]. However, in the present study we do not know how middle managers communicated their need for support from the top management.

The most notable difference among the groups concerned economic incentives, where some PHC managers valued financial incentives and saw them as supporting prioritisation. The HCPs reported no such positive evaluations. Their previous understanding was that economic incentives were less significant in changing health practice, aligning with the conclusion from a Cochrane review [48]. We have not explored the differences in how the participants viewed financial incentives, but it could be related to managers’ thoughts about how to lead and their prerequisites for implementing a leadership supporting evidence-based practice [49]. Other aspects that may increase inner motivation were increased knowledge and competence, all HCPs participating in the change, and manager support with goals, follow-ups, and rewards. These factors were considered of more importance for changing to a practice that would benefit the patients in the long term.

All participants indicated the problem of tight working schedules in PHC. In addition, HCPs felt burdened with heavy workloads and staff shortages. Similar time constraints have been reported as barriers to health promotion interventions in PHC [11,14,15,27]. These time constraints conflicted with the participants’ intentions to promote healthy lifestyles. However, the HCPs had creative suggestions on changes in working schedules and how they could re-organise their routine practice towards a more health-promoting and patient-centred approach. The HCPs’ autonomy could be strengthened by providing opportunities to share ideas, best practices, and knowledge. Yet, the HCPs require education and training to accomplish this practice change [11,50]. They also need the opportunity to create inter-professional teams and to become experts in lifestyle interventions. The participants described a shared vision of PHC, implying similar perceptions of what needs to be done and how they can support each other.

Moreover, the HCPs should be mandated to overcome barriers and solve problems in their daily work. Engaging stakeholders across different levels, including the providers, is recommended [51]. Nilsen et al. recently concluded that organisational changes in health care are more likely to succeed when HCPs can influence the change, be fully prepared for the change, and recognise the value of the change, including that the change will benefit their patients [52]. The support of IFs and EFs was necessary to transform into a health-promoting practice. However, further research is needed on facilitation from the perspectives of IFs and EFs to understand the facilitation process and its contribution to successful implementation and change of practice [53]. We have explored the recipients’ perceptions of barriers, facilitating factors, and the need for support before the implementation. We are using this information to select and adapt the implementation strategy. Whether this contributes to a successful implementation strategy requires evaluation at different levels and implementation stages using theory-driven process evaluation [54].

## Strengths and limitations

This study included 26 participants in focus groups or individual interviews. Transferability was strengthened by including interviewees (HCPs and PHC managers) who varied in age, health care profession and working experience. Most individual interviews were conducted digitally or by phone. Phone interviews have been considered inferior to face-to-face interviews and a study limitation [55]. Still, several studies have suggested the convenience and methodological strengths of conducting qualitative interviews by phone [56,57]. All three authors (two women and one man), with different professional backgrounds (physiotherapist, nurse) but all experienced qualitative researchers, participated in the data analysis. Dependability was strengthened by a structured approach for data analysis, using the CFIR theoretical framework [25] for the deductive analysis. Principles of inductive content analysis were used in the categorisation to gain deeper insight into what the participants said and to detect similarities and differences in statements between the three groups. During this study, an updated version of the CFIR was published [58], where it is concluded that updated constructs can be mapped back to the original CFIR [58]. In this study the statements related to the recipients’ perceived need for support to implement the health-promoting practice were primarily coded into the CFIR constructs: Readiness for Implementation and Implementation Climate within the Inner Setting. These constructs have been removed in the updated CFIR and reclassified as antecedent assessments between implementation determinants and outcomes [58,59]. One reason for removing the constructs was the ‘nesting’ of sub-constructs [58]. In this study, as well as in previous studies [60], it was difficult to distinguish between some sub-constructs, and the analysis required continuing discussions among the authors. The founders of CFIR have also declared that boundaries are fuzzy and dynamic between the domains and constructs, and sometimes difficult to distinguish from another [37]. Still, we consider the CFIR as an appropriate framework for our study aim to explore the need for support for implementation efforts [61]. A strength and novelty of this study is the use of the CFIR before implementation to address the identified contextual determinants at the participating PHC centres to inform, adapt, and prioritise implementation strategies. In addition, the CFIR constructs align well with what is considered important in leading change theories [21,22].

## Conclusion

Primary health care managers, appointed internal facilitators, and health care professionals shared similar expectations on support, implying a mutual view of what would enable them to change into a health promotion practice that benefits their patients. From all levels (i.e. from health care professionals to managers), strong leadership support and engagement were regarded as prerequisites. The support should include assistance in prioritising health promotion from top management and enable the primary health care centres to build competence and take responsibility for the implementation.

## Data Availability

The datasets generated and analysed during the current study are not publicly available because they contain information that could compromise the integrity of the participants but are available from the corresponding author at reasonable request.

## Appendix A. Interview guides on expectations of support

**Table A1.** Interview guide for interviews with managers.

Interview question	CFIR domain, construct and sub-construct
How would you describe the engagement of higher manager levels?	Inner setting – Readiness for Implementation Leadership Engagement
What driving forces would motivate you to lead this work and be a part its success?	Inner setting – Implementation Climate Organizational Incentives and Rewards
How will the health-promotion practice be handled among other, competing, missions and tasks?	Inner setting – Implementation Climate Relative Priority
What changes are needed to implement the practice at the PHC centre?	Inner Setting – Structural Characteristics or Available resources
Do you have a plan for the implementation at your PHC centre?	Implementation Process – Planning, Engaging
What do you expect your employees to do?	Inner setting – Readiness for Implementation Leadership Engagement
What do you think your employees may need to start working according to the health-promotion practice?	Inner setting – Readiness for Implementation Leadership Engagement
How will you engage your employees?	Inner setting – Readiness for Implementation Leadership Engagement
What kind of support may you need?	Inner setting – Readiness for Implementation Leadership Engagement

**Table A2.** Interview guide for interviews with internal facilitators.

Interview question	CFIR domain, construct and sub-construct
Your mission as an internal facilitator is to support your colleagues at the PHC centre, how confident do you feel?	Implementation Process – Formally Appointed Internal Implementation Leaders
How will you start?	Implementation Process – Formally Appointed Internal Implementation Leaders
What kind of support do you think you will have to provide to your colleagues?	Implementation Process – Formally Appointed Internal Implementation Leaders
How do you think your manager will value your efforts as internal facilitator?	Inner setting – Implementation Climate Organizational Incentives and Rewards
What kind of support do you think you will need to accomplish your mission as an internal facilitator?	Implementation Process – Formally Appointed Internal Implementation Leaders
What may trigger your colleagues to work more health-promoting?	Implementation Process – Engaging
What changes might be needed so that you and your colleagues can work according to this health-promoting practice?	Inner Setting – Structural Characteristics or Available resources
How will this health-promoting practice be prioritised among other, competitive, work tasks?	Inner setting – Implementation Climate Relative Priority

**Table A3.** Interview guide for focus group discussions with health care professionals.

Interview question	CFIR domain, construct and sub-construct
What would motivate you to work more health promoting according to this practice?	Characteristics of individuals – Knowledge and Beliefs about the Intervention
How confident do you feel in your ability to implement this practice?	Characteristics of individuals – Self-efficacy
What would trigger you to ask your patients about their lifestyle habits and health?	Implementation Process – Engaging
How will this health-promoting practice be prioritised among other, competitive, work tasks?	Inner setting – Implementation Climate Relative Priority
What would facilitate for you to work according to this health-promoting practice?	Implementation Process – Engaging
What changes might be needed so that you and your colleagues can work according to this health-promoting practice?	Inner Setting – Structural Characteristics or Available resources
What kind of support to do expect from your manager or others to succeed with this?	Inner setting – Readiness for Implementation Leadership Engagement

## Appendix B. Coding matrix with CFIR constructs, general definitions and adapted definitions for the act in time-study

**Table:**

CFIR domain and construct	CFIR general definition construct	CFIR sub-construct	CFIR general definition sub-construct	Adapted definition	Category^a^
Inner Setting					
Readiness for Implementation	Tangible and immediate indicators of an organizational commitment to its decision to implement an innovation.	Leadership Engagement	Commitment, involvement, and accountability of leaders and managers with the implementation of the innovation.	Commitment, involvement, and accountability of PHC middle and top managers with the implementation of the health promoting practice.	Show manager support and clear communication Engage by communicating vision and goals State mandatory to participate
		Available Resources	The level of resources organizational dedicated for implementation and on-going operations including physical space and time	The level and type of present or absent resources in PHC for implementation of the health promoting practice, including staffing and time and how to overcome these barriers.	To have flexible working schedules would overcome time barriers
		Access to Knowledge and Information	Ease of access to digestible information and knowledge about the innovation and how to incorporate it into work tasks.	Ease of access to digestible information and knowledge about health promotion needed to develop and integrate it into work tasks.	Clarify why the change is needed and let us improve our competence Practical support
Implementation Climate	The absorptive capacity for change, shared receptivity of involved individuals to an innovation, and the extent to which use of the innovation will be rewarded, supported, and expected within their organization.	Relative Priority	Individuals’ shared perception of the importance of the implementation within the organization.	PHC managers and HCPs’ perceptions of the importance of the implementation of a health promoting practice within PHC .	Clarify goals and commissions on health promotion Top management supporting high priority of health promotion
		Organizational Incentives and Rewards	Extrinsic incentives such as goal-sharing, awards, performance reviews, promotions, and raises in salary, and less tangible incentives such as increased stature or respect	Extrinsic incentives such as goal sharing, awards, rewards, financial incentives, and less tangible incentives.	Pros and cons of economic incentives Need for follow-ups and rewards
Process					
Engaging	Attracting and involving appropriate individuals in the implementation and use of the innovation through a combined strategy of social marketing, education, role modelling, training, and similar activities.	Formally Appointed Internal Implementation Leaders	Individuals from within the organization who have been formally appointed with responsibility for implementing an innovation as coordinator, project manager, team leader, or other similar role.	Individuals from within the PHC centres who have been formally appointed with responsibility for implementing the health promoting practice at their PHC centre as an internal facilitator.	Appointment of internal facilitators and their role
		External Change Agents	Individuals who are affiliated with an outside entity who formally influence or facilitate innovation decisions in a desirable direction.	Individuals who are affiliated with an outside entity who formally support the PHC centres and facilitate the implementation of the health promoting practice in a desirable direction.	Support to engage co-workers at the PHC centres

PHC: Primary health care.

^a^ Categories were created within the CFIR constructs following the principles of deductive content analysis [36].
